# Risk Assessment and Risk Reduction of Ptaquiloside in Bracken Fern

**DOI:** 10.3390/toxics11020115

**Published:** 2023-01-24

**Authors:** Min Kook Kim, Ji Soo Kang, Amit Kundu, Hyung Sik Kim, Byung-Mu Lee

**Affiliations:** 1 Division of Toxicology, College of Pharmacy, Sungkyunkwan University, 2066 Seobu-ro, Jangan-gu, Suwon-si 16419, Republic of Korea; 2 Laboratory of Molecular Toxicology, College of Pharmacy, Sungkyunkwan University, 2066 Seobu-ro, Jangan-gu, Suwon-si 16419, Republic of Korea

**Keywords:** ptaquiloside, bracken fern, risk assessment, risk reduction, margin of exposure

## Abstract

This study was conducted to determine the optimal boiling time to reduce ptaquiloside (PTA) and to carry out a risk assessment for PTA, a representative toxic substance found in bracken fern (BF; *Pteridium aquilinum*), which is frequently consumed as food in East Asian countries. High-performance liquid chromatography showed that the concentration of PTA in BF was reduced by up to 99% after boiling for 20 min. Risk assessment results showed that the cancer margin of exposure (MOE; ≥ 25,000 = safe) to PTA for an average daily exposure scenario after boiling BF for 20 min was considered safe. In addition, the non-cancer MOE (≥ 300 = safe) to PTA under an average daily exposure scenario after BF boiling for 20 min was considered safe. However, human exposure to PTA was considered unsafe under the non-boiled BF exposure and maximum daily exposure scenarios. Therefore, boiling BF for at least 20 min is recommended before consumption, to reduce exposure to PTA as much as possible.

## 1. Introduction

**Figure 1:**
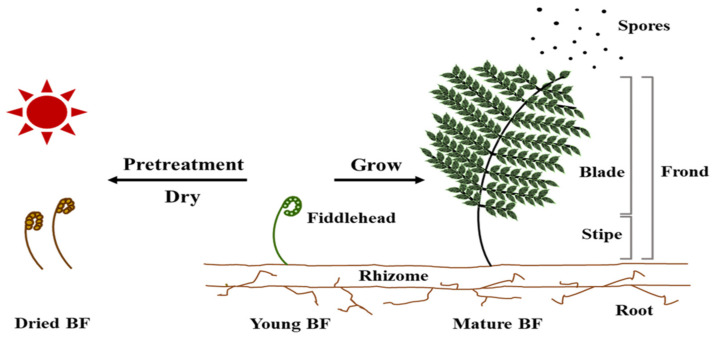
The structure and fate of the bracken fern (BF; adapted from [5]).

Ferns are a group of plants of considerable interest [1], which are less studied than vascular plants for several scientific aspects, such as their taxonomy [2], ecology, habitat, and vegetation [3]. Bracken fern (BF; *Pteridium aquilinum*) is a terrestrial plant with a wide variety of species distributed worldwide [4]. The plants’ structure is divided into roots, rhizomes, and fronds, and immature plants have fiddleheads on the fronds [5] (Figure 1). The fiddlehead gradually spreads out as the BF grows, and after it matures, it spawns and spreads the spores necessary for reproduction [5] (Figure 1). BF is often consumed in conditions of food scarcity by livestock grazing in large areas [6,7], and is an indicator of the pasture presence. By contrast, BF is frequently consumed in stir-fries, soups, and stews in East Asian countries, including Korea, Japan, and China [8,9]. In particular, in Korea, young BF is generally stored after pretreatment and sun-drying, and is then consumed after processing, according to various recipes (Figure 1).

However, BF can be poisonous and may cause esophageal and gastric cancers in humans [9,10,11]. As an example, in a case-control study on 98 patients with esophageal cancer and 476 controls, relative risks of 2.68 (95% confidence interval (CI) = 1.38–5.21) for a daily-BF-intake group and 1.53 (95% CI = 0.90–2.62) for an occasional-BF-intake group were observed, compared to a group with little or no BF intake [12,13,14]. In addition, animal experiments have shown that BF, processed BF, and BF extracts may result in leukemia and intestinal, lung, gastric, bladder, and mammary cancers in various animals, including rodents and cattle [7,9,12,13,15]. These results indicate the carcinogenicity of BF; however, the International Agency for Research on Cancer (IARC) stated that existing human studies had limitations, such as a lack of study details and a lack of consideration of cancer factors other than BF, and that the respective animal studies were conducted with small sample sizes and were reported incompletely [12,13]. Therefore, the IARC classified BF as IARC Group 2B (i.e., possibly carcinogenic to humans) based on intestinal and bladder cancers commonly observed in various animals [12,13].

**Figure 2:**
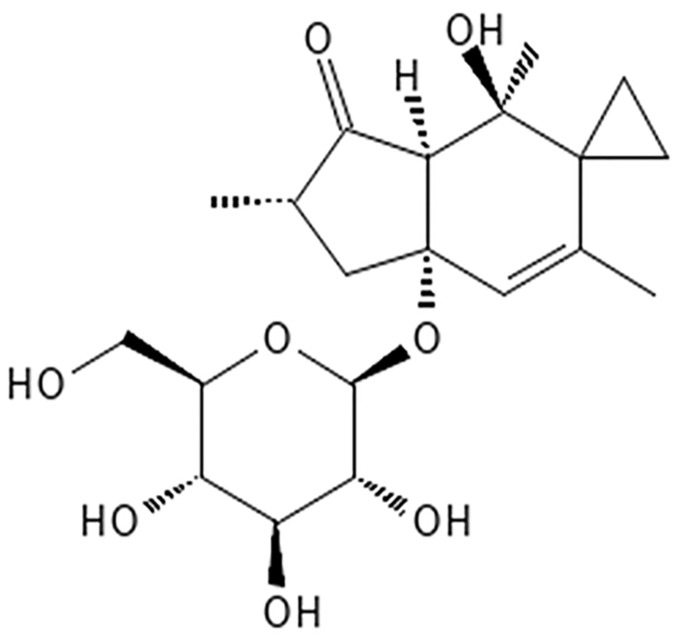
Structural formula of ptaquiloside (PTA).

Potentially carcinogenic BFs contain various toxic substances, including ptaquiloside (PTA), shikimic acid, and thiaminase [9,15,16]. PTA is a norsesquiterpene glucoside and is considered a representative carcinogen in BF [7,9] (Figure 2). PTA can be converted into an unstable intermediate termed ptaquilodienone by the loss of glucose [7], and its epoxide structure can cause point mutations by alkylating selected deoxyribonucleic acid (DNA) bases in a specific codon [7,10]. Therefore, PTA has been reported to induce the activation of the Harvey rat sarcoma viral oncogene homolog (H-ras) [7,17,18]. Activation of H-ras is associated with abnormal cell proliferation, differentiation, and survival [19], thus BF likely elicits carcinogenic effects through PTA [7,17,20].

PTA is observed in the whole tissue of BF, low in roots and spores, and high in edible parts, crosiers [21,22]. Fortunately, PTA is degraded to a stable form by hydrothermal methods [23,24,25] and is expected to exhibit reduced toxicity. However, there is a lack of specific information to prove the reduction of toxicity (i.e., in vitro cytotoxicity test) [26] and degradation conditions such as temperature and heating time. Therefore, this study was conducted to identify the optimal boiling time to remove PTA in BF and to carry out a risk assessment by employing the concentration of PTA analyzed using high-performance liquid chromatography (HPLC). Non-cancer and cancer risk assessments for PTA in the BF were performed according to the European Food Safety Authority (EFSA) guidance [27,28,29].

## 2. Materials and Methods

### 2.1. Chemicals

PTA was purchased from Haihang Industry (Jinan, Shandong, China), and diclofenac sodium, used as an internal standard, was purchased from Sigma-Aldrich (St. Louis, MO, USA). The solvents used for HPLC analysis were of HPLC grade: acetonitrile (ACN) and methanol were purchased from J. T. Baker (Avantor, Radnor, PA, USA), and HPLC water was purchased from Sigma-Aldrich (St. Louis, MO, USA).

### 2.2. Pretreatment and Sampling of BF for HPLC Analysis

Twenty types of dried BF were purchased from retail stores in Korea. Dried BF was soaked in water for 4 h to obtain conditions similar to those of typical preparation for consumption. Subsequently, BF was boiled for 10 or 20 min or not boiled.

For HPLC sample preparation, 10 g of non-boiled or boiled BF was ground in 10 mL HPLC water and was centrifuged twice for 5 min at 3500 rpm. The supernatant was then filtered through a 0.2-μm Acrodisc syringe filter (Pall, Port Washington, NY, USA), and 200 μL was mixed with the same amount of ACN. The mixture was vortexed for 3 min and was centrifuged for 10 min at 5000 rpm. Then, 150 μL supernatant was mixed with 50 μL HPLC water to prepare 200 μL of the final HPLC sample. All sampling processes were performed at pH 7 ± 0.2 as PTA is stable at neutral pH [25].

### 2.3. HPLC Analysis Conditions

**Figure 3:**
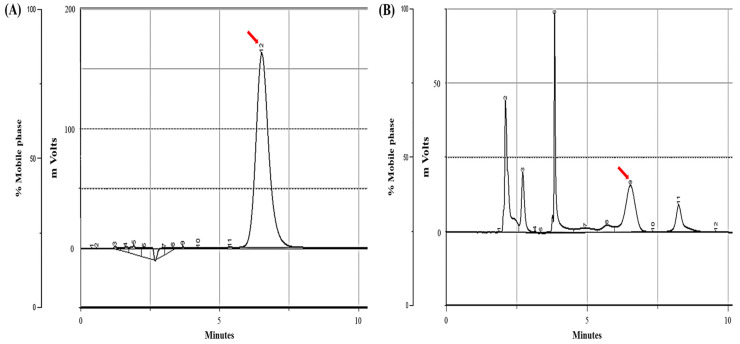
High-performance liquid chromatography (HPLC) chromatograms of PTA. (**A**) PTA (200 μg/mL) standard sample (arrow) and (**B**) BF sample (arrow).

Calibration plots were constructed from a standard sample spiked with an internal standard and eight PTA concentrations (1.56–200 μg/mL). The ultraviolet wavelength of PTA measured before HPLC analysis was 220 nm, and HPLC was performed using a Gilson (Middleton, WI, USA) instrument equipped with a C18 column (Synergi 4 µm Hydro-RP 80 Å, LC Column 250 × 4.6 mm; Phenomenex, Torrance, CA, USA). The HPLC samples were transferred at a flow rate of 0.8 mL/min at room temperature with an isocratic mobile phase consisting of a mixture of water and methanol at a ratio of 40:60 *v*/*v*, and the retention time of PTA was confirmed to be 6.68 min (Figure 3). The calibration curve was produced through weighted (1/x) least-squares linear regression, and the coefficient of determination (r^2^) for the calibration curve was 0.9983. The limit of detection of PTA was 0.05 ppm.

### 2.4. Human Exposure to PTA

Human exposure to PTA was calculated by considering daily BF intake, PTA concentration, oral absorption rate, and body weight, according to the following equation:

Human exposure (mg/kg/day) = (daily BF intake (g/day) × 1000 × concentration of PTA (%)/100 × oral absorption rate (%)/100) ÷ body weight (kg)(1)

Daily BF intake was determined to be 2.32 g/day, corresponding to the average of the total population according to statistical data reported in Korea [30], and the concentration of PTA was determined as the median and maximal values of the analyzed concentrations to represent average and maximal exposure scenarios. The oral absorption rate was not reported previously; thus, it was assumed to be 100%, and the body weight was considered to be 70 kg, the default value for adults [27].

### 2.5. Non-Cancer and Cancer Risk Assessment

Risk assessment for PTA in BF was conducted based on the EFSA guidance [27,28,29]. In general, the non-cancer risk assessment results are expressed as the margin of exposure (MOE) = no observed adverse effect level (NOAEL)/human exposure, and if the MOE exceeds 100, it is considered safe [28]. However, if the lowest observed adverse effect level (LOAEL) is used instead of NOAEL, an uncertainty factor of three can be added [27]. By contrast, the cancer risk assessment results are expressed as the benchmark dose lower confidence limit for a 10% response (BMDL_10_)/human exposure, and if the MOE is greater than 10,000, it is considered safe [28]. However, if the data are not suitable for estimating BMDL_10_ (e.g., single linear model) [31], a chronic dose that causes tumors in a certain tissue region in 25% of the experimental animals after the rate is corrected with the spontaneous carcinogenesis rate (T25) can be used, and an uncertainty factor of 2.5 can be added [28,29,32].

## 3. Results

### 3.1. Determination of Optimal Boiling Time for Removal of PTA from BF

The optimal boiling time was investigated using five soaked BF batches. The concentration of PTA in BF boiled for 10 min was 10.82–33.61 ppm, which was decreased by 70.14–88.26% compared to non-boiled BF (Table 1). The concentration of PTA in BF boiled for 20 min was 2.48–6.54 ppm, which was reduced by 94.19–97.31% compared to the non-boiled BF (Table 1). The concentration of PTA in BF decreased as boiling time increased (Table 1), and 20 min of boiling with a PTA reduction rate of at least 94% was determined to be the appropriate boiling condition.

### 3.2. Concentration of PTA after Boiling BF for 20 Min

The PTA concentration was measured using 20 soaked BF samples. The concentration of PTA in non-boiled BF was 12.50–1995.38 ppm, with a median of 409.55 ppm (Table 2). The concentration of PTA in BF boiled for 20 min was 2.31–132.74 ppm, with a median of 5.78 ppm (Table 2). The concentration of PTA in BF thus decreased by 79.61ȁ99.24% after boiling, with a median decrease of 95.51% (Table 2).

### 3.3. Human Exposure to PTA

Human exposure to PTA was calculated for average and maximum exposure scenarios. Human exposure to PTA was 1.36 × 10^−2^ mg/kg/day for non-boiled BF and 1.92 × 10^−4^ mg/kg/day for BF boiled for 20 min in the average exposure scenario (Table 3). Under the maximum exposure scenario, human exposure to PTA was 6.63 × 10^−2^ mg/kg/day for non-boiled BF and 4.41 × 10^−3^ mg/kg/day for BF boiled for 20 min (Table 3).

### 3.4. Determination of Non-Cancer and Cancer Toxicity Values for PTA

The toxicity values for PTA were derived from the most appropriate study on PTA toxicity [14]. The non-cancer toxicity value was derived from a different study [33], and the cancer toxicity value was derived from a further study [34]. The details of the selected toxicity data and methods for calculating toxicity values are summarized in Table 4.

### 3.5. Non-Cancer and Cancer Risk Assessment for PTA

The results of the non-cancer and cancer risk assessments were derived by calculating the MOE. The non-cancer MOE was 7.65 for non-boiled BF and 541.67 for BF boiled for 20 min for the average exposure scenario (Table 5). The cancer MOE was 415.59 for non-boiled BF and 29,437.50 for BF boiled for 20 min (Table 5). Under the maximal exposure scenario, the non-cancer MOE was 1.57 for non-boiled BF and 23.58 for BF boiled for 20 min (Table 5). The cancer MOE was 85.25 for non-boiled BF and 1281.63 for BF boiled for 20 min (Table 5). It was thus considered unsafe, except for the non-cancer and cancer risk assessment results for BF boiled for 20 min under the average exposure scenario (Table 5).

## 4. Discussion

Plants contain various substances, including toxic compounds [36,37,38], and risk assessment of these substances is considered important for risk management [39,40,41,42,43]. The present study is the first to conduct non-cancer and cancer risk assessments of PTA in BF. For the risk assessment, the concentration of PTA in BF was measured using HPLC, and the toxicity value of PTA was derived from animal toxicity data. In addition, human exposure to PTA was calculated according to an equation, and the EFSA guidance was referred to [27,28,29].

The concentration of PTA in BF decreased as boiling time increased (Table 1), and the reduction rate of PTA after 20 min of boiling was determined as the optimal boiling condition was up to 99.24% (Table 2). These results were compared with those of a previous study [23], which predicted the concentration of PTA in BF using pterosin B (PTB), a stable metabolite of PTA; this previous study showed that the PTB reduction rates for raw BF boiled for 5 and 10 min were 60% and 66.4%, respectively. In addition, the reduction rate of PTB was 88% for raw BF soaked in water for 12 h after boiling for 5 min [23]. These results indicated that the concentration of PTA in BF decreased with boiling time, which was in line with our results (Table 1), and that it also decreased with soaking time and the number of water exchanges. This explains why BF is typically soaked in water and boiled before consumption.

Human exposure to PTA was calculated using a formula and was found to be lower for BF boiled for 20 min than for non-boiled BF (Table 3). Our results were compared with those of human exposure to PTA in drinking water as examined in a previous study [14], where the 99th percentile lifetime average human exposure was calculated as 0.5 μg/kg/day considering the maximal exposure scenario. This value was 132.6-fold and 8.82-fold lower than that for non-boiled BF and BF boiled for 20 min, respectively, under the maximal exposure scenario (Table 3). Comparative results showed that drinking water is not the main route of human exposure to PTA and that dietary exposure is more prevalent [14]. In addition, humans can be exposed to PTA through milk and soil [7,44,45].

The toxicity values for PTA, LOAEL, and T25 were derived from animal toxicity data, however, these data contained limited information (Table 4). Non-cancer and cancer studies were conducted with only one variable dose group for a small number of animals, and the cancer study was a 207-day study rather than a two-year study (Table 4). However, this result was expected because the concentration of PTA in BF is not constant, and PTA is unstable [33,34].

The risk assessment results showed that human exposure to PTA is only safe for BF boiled for 20 min under the average exposure scenario for non-cancer and cancer risk assessment (Table 5). These results may be because exposure was assessed using an oral absorption rate of 100% due to the lack of data. However, human exposure to PTA may require caution under a conservative risk assessment approach that considers a conservative assessment factor and a maximal exposure scenario. Similar to our results, when non-cancer and cancer risk assessments were evaluated using human exposure to PTA through 0.5 μg/kg/day of drinking water [14], it was calculated as non-cancer MOE 208 and cancer MOE 11,304 and was considered unsafe.

PTA is a toxic substance contained in BF that can be readily absorbed by consumers through the diet. Therefore, it is suggested that BF should be sufficiently boiled, i.e., for at least 20 min, before consumption to reduce human exposure to PTA. In addition, further studies, in addition to the proposed method, are needed to avert human exposure to PTA. Finally, in future studies, it seems necessary to establish a database for optimal risk reduction conditions for each food containing toxic substances.

## Figures and Tables

**Table 1:** Analysis of the concentration of PTA in BF according to boiling time.

Sample	Concentration of PTA ^1^ (Reduction Rate of PTA)		
	Soaked BF ^2^	BF Soaked ^2^ and Then Boiled for 10 Min	BF Soaked ^2^ and Then Boiled for 20 Min
1	112.58 ppm	33.61 ppm (70.14%)	6.54 ppm (94.19%)
2	108.20 ppm	29.07 ppm (73.14%)	4.72 ppm (95.64%)
3	84.95 ppm	16.23 ppm (80.89%)	3.93 ppm (95.37%)
4	92.15 ppm	10.82 ppm (88.26%)	2.48 ppm (97.31%)
5	107.27 ppm	24.63 ppm (77.04%)	4.03 ppm (96.24%)
Range	84.95–112.58 ppm	10.82–33.61 ppm (70.14–88.26%)	2.48–6.54 ppm (94.19–97.31%)

^1^ Limit of detection = 0.05 ppm. ^2^ Dried BF was soaked in water for 4 h.

**Table 2:** Analysis of the concentration of PTA in BF after boiling for 20 min.

Sample	Concentration of PTA (ppm) ^1^		Reduction Rate of PTA (%)
	Soaked BF ^2^	BF Soaked ^2^ and Then Boiled for 20 Min	
1	112.58	6.54	94.19
2	108.20	4.72	95.64
3	84.95	3.93	95.37
4	92.15	2.48	97.31
5	107.27	4.03	96.24
6	12.50	2.31	81.51
7	45.22	9.22	79.61
8	168.20	32.06	80.94
9	137.22	12.28	91.05
10	563.84	50.93	90.97
11	391.54	9.39	97.60
12	1303.15	132.74	89.81
13	1995.38	39.87	98.00
14	433.35	88.05	79.68
15	540.09	4.21	99.22
16	476.59	5.01	98.95
17	461.96	4.07	99.12
18	489.34	27.17	94.45
19	427.56	3.36	99.21
20	518.02	3.94	99.24
Range	12.50–1995.38	2.31–132.74	79.61–99.24
Median	409.55	5.78	95.51

^1^ Limit of detection = 0.05 ppm. ^2^ Dried BF was soaked in water for 4 h.

**Table 3:** Human exposure to PTA.

Daily BF Intake (g/Day) ^1^	Concentration of PTA (%)			Oral Absorption Rate (%) ^3^	Body Weight (kg) ^4^	Human Exposure (mg/kg/Day) ^5^
2.32	Average exposure (median PTA value)	Soaked BF ^2^	4.10 × 10^−2^	100	70	1.36 × 10^−2^
		BF soaked ^2^ and then boiled for 20 min	5.78 × 10^−4^			1.92 × 10^−4^
	Maximal exposure (maximal PTA value)	Soaked BF ^2^	2.00 × 10^−1^			6.63 × 10^−2^
		BF soaked ^2^ and then boiled for 20 min	1.33 × 10^−2^			4.41 × 10^−3^

^1^ Average daily intake across the total population of Korea [30]. ^2^ Dried BF was soaked in water for 4 h. ^3^ 100%; assumed due to lack of data. ^4^ Assumed a default adult value of 70 kg [27]. ^5^ Human exposure (mg/kg/day) = (daily BF intake (g/day) × 1000 × concentration of PTA (%)/100 × oral absorption rate (%)/100) ÷ body weight (kg). Abbreviation: BF, bracken fern; PTA, ptaquiloside.

**Table 4:** Determination of non-cancer and cancer toxicity values for PTA.

Toxicity Endpoint	Animal Species	Duration	Dose	Toxicity Value	Reference
Non-cancer	Rat	90 days	- 4 controls: normal diet - 6 experimental animals: diet containing 25% dried BF (PTA 4.6–20.7 mg/kg in BF) = PTA 1.15–5.175 mg/kg in diet = PTA 0.104–0.466 mg/kg/day ^1^	LOAEL: 0.104 mg/kg/day ^3^ - Decrease in body weight - Increase in spleen weight - Decrease in the number of red blood cells, white blood cells, and lymphocytes in the blood - Decrease in glucose and increase in AST and ALT in serum	[33]
Cancer	Rat	207 days	- 20 controls: normal diet - 15 experimental animals: diet containing PTA (0.04% for 15 days, 0.027% for 40 days, 0.04% for 52 days, 0.08% for 40 days, and 0.04% for 60 days) = PTA 0.0452% (452.174 mg/kg) in diet = PTA 22.609 mg/kg/day ^2^	T25: 5.652 mg/kg/day ^4^ - Observation in ileal and bladder tumors (controls: 0/20 and experimental animals: 15/15)	[34]

^1^ Calculated using a subchronic conversion factor (conversion of the concentration of substance in the feed (mg/kg) to the daily dose (mg/kg/day)) of 0.09 for rat [27]. ^2^ Calculated using a chronic conversion factor (conversion of the concentration of substance in the feed (mg/kg) to the daily dose (mg/kg/day)) of 0.05 for rat [27]. ^3^ Determined as 0.104 mg/kg/day considering the conservative value. ^4^ Calculated according to the T25 calculation method of [35] (22.609 mg/kg/day × 25%). Abbreviations: ALT, alanine transaminase; AST, aspartate transaminase; BF, bracken fern; LOAEL, lowest observed adverse effect level; T25, chronic dose that causes tumors in a certain tissue region in 25% of experimental animals after the rate is corrected with the spontaneous carcinogenesis rate.

**Table 5:** Non-cancer and cancer risk assessment results for PTA.

Toxicity Value (mg/kg/Day) ^1^		Human Exposure (mg/kg/Day) ^2^			Risk Assessment Result	
Non-Cancer	Cancer				Non-Cancer MOE (≥300 = safe) ^4^	Cancer MOE (≥25,000 = safe) ^5^
LOAEL: 0.104	T25: 5.652	Average exposure (median PTA value)	Soaked BF ^3^	1.36 × 10^−2^	7.65 (not safe)	415.59 (not safe)
			BF soaked ^3^ and then boiled for 20 min	1.92 × 10^−4^	541.67 (safe)	29,437.50 (safe)
		Maximal exposure (maximal PTA value)	Soaked BF ^3^	6.63 × 10^−2^	1.57 (not safe)	85.25 (not safe)
			BF soaked ^3^ and then boiled for 20 min	4.41 × 10^−3^	23.58 (not safe)	1281.63 (not safe)

^1^ (Table 4). ^2^ (Table 3). ^3^ Dried BF was soaked in water for 4 hr. ^4^ Non-cancer MOE = LOAEL/human exposure (MOE ≥ 300 = safe), an additional safety factor of 3 to the safe criteria MOE 100 was employed for extrapolation of LOAEL to NOAEL [27,28]. ^5^ Cancer MOE = T25/human exposure (MOE ≥ 25,000 = safe), an additional safety factor of 2.5, to the safe criteria MOE 10,000 was employed to replace BMDL_10_ with T25 [28,29,32]. Abbreviations: BF, bracken fern; BMDL_10_, benchmark dose lower confidence limit for a 10% response; LOAEL, lowest observed adverse effect level; MOE, margin of exposure; NOAEL, no observed adverse effect level; T25, chronic dose that causes tumors in a certain tissue region in 25% of experimental animals after the rate is corrected with the spontaneous carcinogenesis rate.

## Data Availability

Data is contained within the article.
